# Two case reports of continued progression of chronic ocular graft-versus-host disease without concurrent systemic comorbidities treated by amniotic membrane transplantation

**DOI:** 10.1186/s12886-021-01925-3

**Published:** 2021-04-07

**Authors:** Hideto Ikarashi, Naohiko Aketa, Eisuke Shimizu, Yoji Takano, Tetsuya Kawakita, Yuichi Uchino, Yukihiro Matsumoto, Junko Ogawa, Kazuo Tsubota, Yoko Ogawa

**Affiliations:** 1 Department of Ophthalmology, Keio University School of Medicine, Tokyo, Japan; 2 Department of Ophthalmology, Kawasaki Municipal Ida Hospital, Kanagawa, Japan; 3 Department of Ophthalmology, Kitasato University Kitasato Institute Hospital, Tokyo, Japan

**Keywords:** Severe ocular GVHD, Dry eye disease, Amniotic membrane transplantation, Fibrosis, Inflammation

## Abstract

**Background:**

Chronic ocular graft-versus-host disease (oGVHD) is an ocular comorbidity of graft-versus-host disease (GVHD) that usually occurs concurrently with systemic manifestations. Failure to detect and treat oGVHD in its early stages may lead to progression of ocular signs and symptoms leading to oGVHD that is refractory to conventional treatment.

**Case presentation:**

We report the clinical course of a 19-year-old male and a 59-year-old female with severe and progressive chronic oGVHD without concurrent systemic signs of chronic graft-versus-host disease (cGVHD). Although their systemic conditions had been stable, both suffered from severe oGVHD and were referred to our clinic. Both cases exhibited marked improvement in conjunctival inflammation and fibrotic changes after amniotic membrane transplantation (AMT). Both cases underwent keratoplasty eventually to stabilize ocular surface conditions and to improve visual function.

**Conclusions:**

We reported the clinical outcomes of 2 cases of chronic oGVHD without concurrent systemic comorbidities that were treated with AMT. The clinician should be aware that cGVHD may persist in target organs even in the absence of concurrent systemic comorbidities following seemingly successful systemic treatment. A multidisciplinary team approach is essential in the early detection and therapeutic intervention for chronic oGVHD.

## Background

Dry eye disease (DED) is the most frequent ocular manifestation after haematopoietic stem cell transplantation (HSCT) [1, 2], and more than half of patients with GVHD-related dry eye rapidly develop severe DED [3], usually occurring concurrently with systemic manifestations [4]. Several treatments have been reported to alleviate mild chronic oGVHD; however, severe oGVHD is intractable to the best available therapies [4]. The characteristic features of severe chronic oGVHD are inflammation and fibrosis, which lead to symblepharon, fornix shortening, limbal stem cell deficiency (LSCD), corneal neovascularization and conjunctivalization [4, 5].

The human amniotic membrane (AM) is reported to exert anti-inflammatory and anti-scarring actions [6–9], and amniotic membrane transplantation (AMT) has been used to treat severe ocular surface diseases such as Stevens-Johnson syndrome and ocular cicatricial pemphigoid. Cultivated oral mucosal epithelial transplantation (COMET) is also a new treatment option for severe oGVHD with limbal stem cell deficiency (LSCD) but is not applicable for cases with concurrent oral GVHD, not readily available due to a need for cell culture, and is more costly than AMT.

In this article, we report the clinical outcomes of 2 cases of refractory chronic oGVHD without long-term systemic comorbidities, both of which were treated with AMT and keratoplasty to achieve clinical improvement.

## Case presentation

### Case 1

**Fig. 1:**
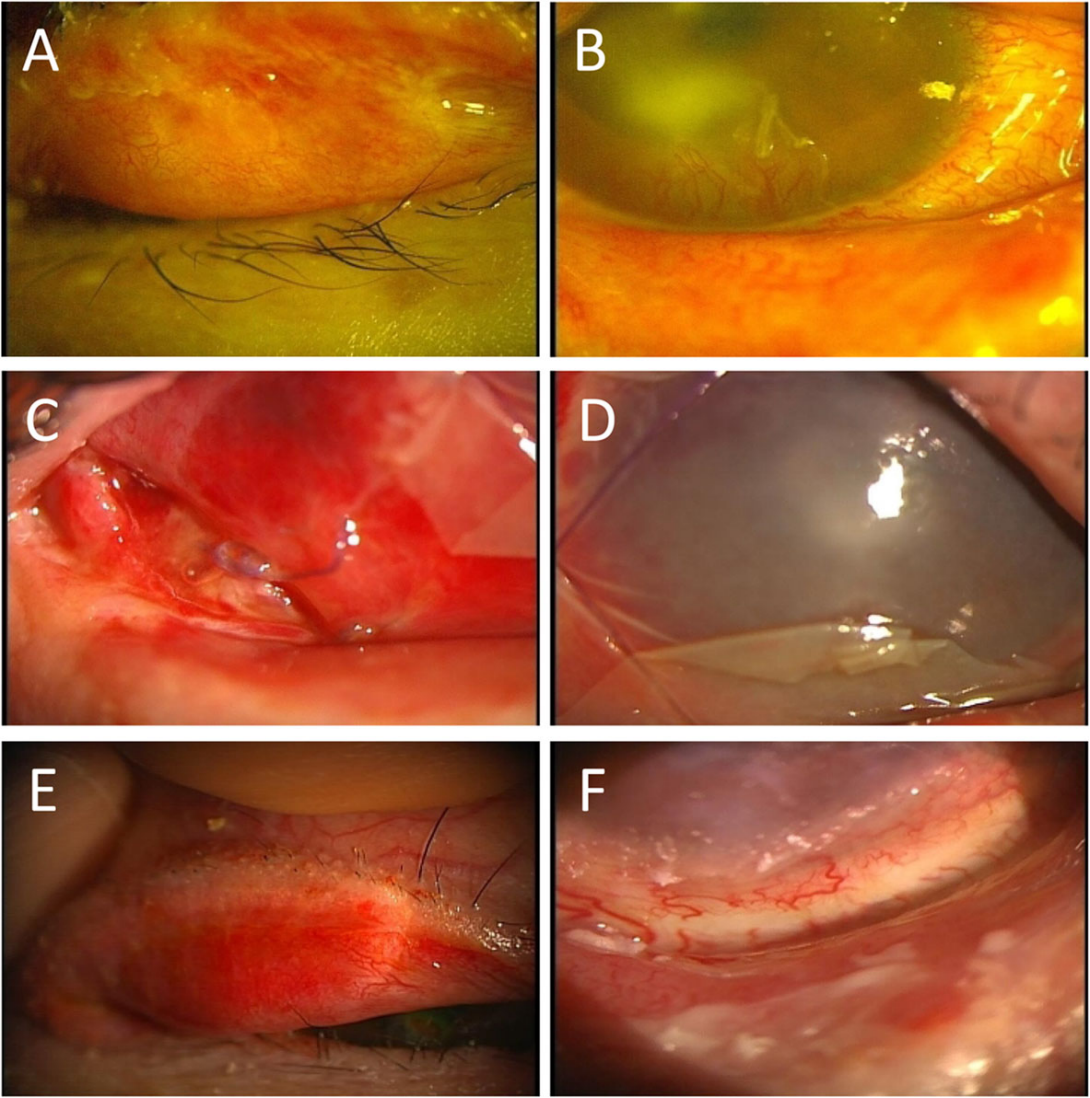
Slit-lamp micrographs before and after amniotic membrane transplantation (AMT) for a 19-year-old male with GVHD-related dry eye disease. **a**, **b** Conjunctival fibrosis and active neovascularization before AMT. **c**, **d** An AM was used to cover the ocular surface, and AM implantation at the site of fornix shortening was performed. **e**, **f** After AMT, tarsal conjunctival fibrosis subsided, and fornix was reconstructed

A 19-year-old Japanese male with a history of acute myelogenous leukaemia (AML) who underwent reduced intensity allogeneic HSCT (mini-transplantation) in March 2005 was referred to the Keio University Dry Eye outpatient clinic in October 2005 complaining of severe ocular pain, difficulty opening his eyes and foreign body sensations after acute and chronic skin GVHD were completely stabilized. Prior to mini-transplantation, no ocular surface abnormality was noted at the previous hospital, and he started to suffer from newly developed symptoms of DED in May 2005, after alleviation of acute skin GVHD. Systemic prednisolone (PSL) had been tapered from 40 to 20 mg/day in July and was discontinued in August 2005. Just after cessation of PSL, bilateral lid swelling and severe ocular pain had emerged. PSL (15 mg/day) was restarted to treat skin chronic GVHD in October 2005. Skin chronic GVHD was well controlled by this treatment. At the first visit to Keio University Dry Eye outpatient clinic in October 2005, severe DED, extensive trichiasis, corneal conjunctivalization, corneal ulcer, spontaneous lacrimal punctal occlusion, and active corneal neovascularization were observed in both eyes (Fig. 1). Best-corrected visual acuity (BCVA) was 20/100 in his right eye and 20/32 in his left eye. Although the patient had been treated with all the available conventional pharmacotherapies, ocular inflammation was not sufficiently controlled. Therefore, we decided to perform AMT on both eyes in October 2005. After releasing the symblephara, the AM was implanted not only onto the surface of the bulbar conjunctiva but also onto the deep fornices. Three months after AMT, the fornices were deep and well maintained, and after 9 months, visual acuity improved to 20/25 in his right eye and 20/22 in his left eye. Contact lens was used to alleviate severe ocular pain due to extensive trichiasis, but a corneal perforation of unknown aetiology on his right eye occurred 1 year after AMT. Penetrating keratoplasty was performed on his right eye without complications, and no rejection has occurred with topical steroid application during the observation period. As of Mar 2021, systemic GVHD progression has also not been observed with low-dose systemic PSL.

### Case 2

**Fig. 2:**
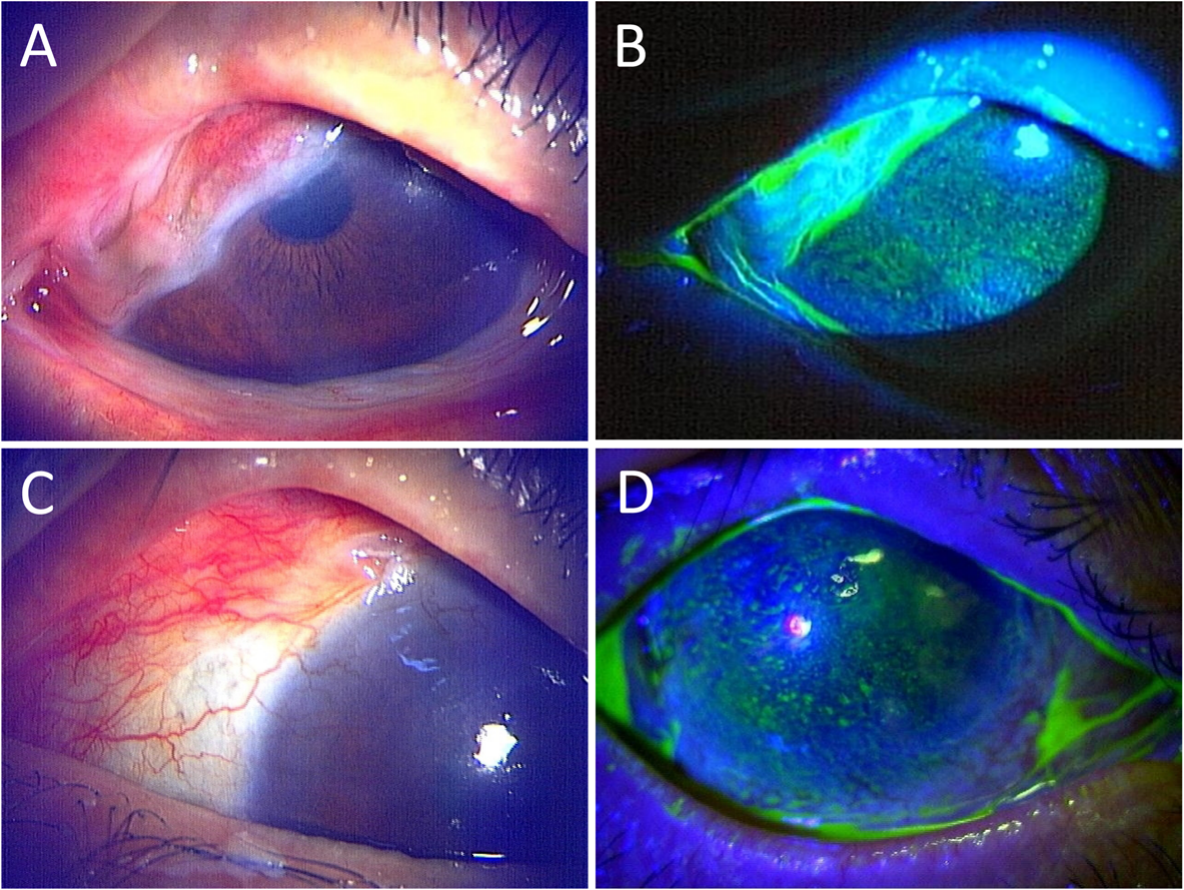
Slit-lamp micrographs before and after amniotic membrane transplantation (AMT) for a 59-year-old female with GVHD-related dry eye disease. **a**, **b** Symblepharon, fornix shortening, conjunctivalization, and neovascularization before AMT. **c**, **d** After AMT, symblepharon and conjunctivalization improved, and fornix was reconstructed. Fluorescein-stained ocular surface images before (**b**) and after (**d**) AMT

A 59-year-old Japanese female with a history of mixed phenotype acute leukaemia (lymphoid 60% and myeloid 40%) who underwent allogeneic bone marrow transplantation (BMT) without irradiation in May 2010 was referred to the Keio University Dry Eye outpatient clinic in September 2013 as she developed refractory chronic oGVHD. After BMT, she developed acute skin GVHD and was successfully treated with systemic tacrolimus (2 mg/day) and PSL (30 mg/day), which was tapered over almost 1 year. In early 2011, just after tapering of treatment for acute skin GVHD, she developed DED and transient difficulty opening her mouth, which was diagnosed as chronic ocular and oral GVHD. She had been suffering from symblepharon, LSCD and conjunctivalization in her right eye, and she had been previously treated with contact lenses, commercially available eye drops, autologous serum and topical cyclosporine and tacrolimus, yet her ocular surface condition had not improved. At the first visit to Keio University Hospital in 2013, severe conjunctival fibrosis of the upper conjunctiva and fornix shortening, partial LSCD and conjunctivalization of the upper cornea on her right eye were observed (Fig. 2) despite no sign of active systemic GVHD. BCVA was 20/100 in her right eye and 20/20 in her left eye. Therefore, we decided to perform AMT on the right eye. Conjunctival fibrotic tissue was removed from the upper fornix to the limbus, and AMT was performed as a substrate and to cover the ocular surface. The patient was able to undergo cataract surgery on the right eye 1 year after AMT as improvement of corneal clarity in the pupillary area of the right eye was achieved. Two years after AMT, there was only a minor recurrence of corneal conjunctivalization, and BCVA was 20/28 in her right eye. She reported that her daily life activity had been satisfactory without any inconvenience for more than 27 months since AMT. However, corneal conjunctivalization of the right eye progressed slowly, and AMT with allogenic cultivated limbal epithelial transplant was performed on the right eye. Her postoperative course has been good for more than 2 years after this procedure, and BCVA has been maintained at 20/50 in her right eye. As of Mar 2021, her ocular surface condition is stable with topical tacrolimus, steroids, and autologous serum. Systemic GVHD progression has not been observed postoperatively with systemic tacrolimus and low-dose PSL.

## Discussion and conclusions

Chronic oGVHD is an immune-mediated fibrotic disease that usually occurs concurrently with systemic comorbidities, and its aetiologies are multifactorial and still largely unknown. Currently available treatments such as eye drops, oral medications or punctal plugs are often insufficient to control severe ocular surface inflammation and excessive fibrosis, and additional therapeutic interventions such as AMT, corneal transplantations, and COMET are indicated for this intractable disease. The availability of such surgical interventions is limited, and the indications for these interventions should be considered carefully. Therefore, it is vital to detect oGVHD in the early phase to prevent severe ocular complications.

AMT has been reported to be effective in treating refractory ocular surface diseases, including Stevens-Johnson syndrome and ocular cicatricial pemphigoid [9, 10]. Clinical manifestations of severe chronic oGVHD, including conjunctival fibrosis and severe ocular inflammation, are similar to those of other diseases. There have been two reports of AMT conducted for corneal perforation in chronic oGVHD [11, 12]. AM is reported to suppress TGF-β signalling and myofibroblast differentiation in cultured human corneal and limbal fibroblasts [8]. AMT was widely disseminated by Tseng SC, et al., and he later reported that the heavy chain hyaluronan/pentraxin 3 (HC-HA/PTX3) derived from the AM had anti-inflammatory and anti-fibrotic effects [13]. We also reported the effectiveness of HC-HA/PTX3 in inhibiting inflammation and the fibrosis of lacrimal glands affected by cGVHD using an established animal model [14].

In both cases the fornix was successfully reconstructed and long-term maintenance was achieved after AMT. However, a minor recurrence of corneal conjunctivalization in case 2 and corneal perforation in case 1 were observed after AMT, and both were treated with corneal keratoplasty.

Although acute skin GVHD in cases 1 and 2 and transient chronic oral GVHD in case 2 were observed, oGVHD continued to progress rapidly even after stabilization of systemic conditions. Both cases resulted in severe oGVHD during or after tapering of systemic corticosteroids or immunosuppressants and AMT was required to reconstruct the fornix and to prevent corneal conjunctivalization due to LSCD.

The limbal stem cell region has been reported to be infiltrated by inflammatory cells based on confocal microscopy observations in chronic oGVHD [15]. Furthermore, it has been reported that intraocular cytokine levels were elevated in chronic ocular surface diseases including oGVHD [16]. We speculate that the ocular surface in these cases had been subclincally inflamed during acute GVHD, and then manifested as severe chronic oGVHD after the cessation of systemic anti-inflammatory treatment.

cGVHD patients are immunocompromised, and monitoring for ocular surface infections as well as systemic conditions is imperative. However, ocular manifestations may become apparent even after stabilization of systemic GVHD, as we experienced in the two cases reported herein. We suspect that these patients had hidden or latent mucosal or exocrine gland GVHD involving several mucosal surface barrier regions. The clinician therefore should be aware of the possibility of subclinical mucosal inflammation in cGVHD and the possibility of exacerbation of such inflammation especially at cessation or tapering of systemic anti-inflammatory medication. A multidisciplinary team approach is essential in determining the optimal timing for tapering of systemic anti-inflammatory medication in order to minimize the risk of chronic oGVHD.

## Data Availability

The data that support the findings of this study are available within the article or from the corresponding author upon reasonable request.

## Abbreviations

AM: Amniotic membrane
AML: Acute myelogenous leukaemia
AMT: Amniotic membrane transplantation
BMT: Bone marrow transplantation
cGVHD: Chronic graft-versus-host disease
COMET: Cultivated oral mucosal epithelial transplantation
DED: Dry eye disease
GVHD: Graft-versus-host disease
LSCD: Limbal stem cell deficiency
HSCT: Haematopoietic stem cell transplantation
oGVHD: Ocular graft-versus-host disease
PSL: Prednisolone

## References

[1] Rapoport Y, Freeman T, Koyama T, Engelhardt BG, Jagasia M, Savani BN, Tran U, Kassim AA (2017). Validation of international chronic ocular graft-versus-host disease (GVHD) group diagnostic criteria as a chronic ocular GVHD-specific metric. Cornea.

[2] Dietrich-Ntoukas T (2015). Clinical signs of ocular graft-versus-host disease. Klin Monatsbl Augenheilkd.

[3] Ogawa Y, Okamoto S, Wakui M, Watanabe R, Yamada M, Yoshino M, Ono M, Yang HY, Mashima Y, Oguchi Y, Ikeda Y, Tsubota K (1999). Dry eye after haematopoietic stem cell transplantation. Br J Ophthalmol.

[4] Shikari H, Antin JH, Dana R (2013). Ocular graft-versus-host disease: a review. Surv Ophthalmol.

[5] Jagasia MH, Greinix HT, Arora M, Williams KM, Wolff D, Cowen EW, Palmer J, Weisdorf D, Treister NS, Cheng GS, Kerr H, Stratton P, Duarte RF, McDonald GB, Inamoto Y, Vigorito A, Arai S, Datiles MB, Jacobsohn D, Heller T, Kitko CL, Mitchell SA, Martin PJ, Shulman H, Wu RS, Cutler CS, Vogelsang GB, Lee SJ, Pavletic SZ, Flowers MED (2015). National Institutes of Health consensus development project on criteria for clinical trials in chronic graft-versus-host disease: I. the 2014 diagnosis and staging working group report. Biol Blood Marrow Transplant.

[6] Fujishima H, Shimazaki J, Shinozaki N, Tsubota K (1998). Trabeculectomy with the use of amniotic membrane for uncontrollable glaucoma. Ophthalmic Surg Lasers.

[7] Takano Y, Fukagawa K, Miyake-Kashima M, Tanaka M, Asano-Kato N, Dogru M, Tsubota K, Fujishima H (2004). Dramatic healing of an allergic corneal ulcer persistent for 6 months by amniotic membrane patching in a patient with atopic keratoconjunctivitis: a case report. Cornea.

[8] Tseng SC, Li DQ, Ma X (1999). Suppression of transforming growth factor-beta isoforms, TGF-beta receptor type II, and myofibroblast differentiation in cultured human corneal and limbal fibroblasts by amniotic membrane matrix. J Cell Physiol.

[9] Tsubota K, Satake Y, Ohyama M (1996). Surgical reconstruction of the ocular surface in advanced ocular cicatricial pemphigoid and Stevens-Johnson syndrome. Am J Ophthalmol.

[10] Honavar SG, Bansal AK, Sangwan VS, Rao GN (2000). Amniotic membrane transplantation for ocular surface reconstruction in Stevens-Johnson syndrome. Ophthalmology.

[11] Peris-Martinez C, Menezo JL, Diaz-Llopis M (2001). Multilayer amniotic membrane transplantation in severe ocular graft versus host disease. Eur J Ophthalmol.

[12] Yeh PT, Hou YC, Lin WC, Wang IJ, Hu FR (2006). Recurrent corneal perforation and acute calcareous corneal degeneration in chronic graft-versus-host disease. J Formos Med Assoc.

[13] Tseng SC (2016). HC-HA/PTX3 purified from amniotic membrane as novel regenerative matrix: insight into relationship between inflammation and regeneration. Invest Ophthalmol Vis Sci.

[14] Ogawa Y, He H, Mukai S, Imada T, Nakamura S, Su CW, Mahabole M, Tseng SCG, Tsubota K (2017). Heavy chain-hyaluronan/pentraxin 3 from amniotic membrane suppresses inflammation and scarring in murine lacrimal gland and conjunctiva of chronic graft-versus-host disease. Sci Rep.

[15] He J, Ogawa Y, Mukai S, Saijo-Ban Y, Kamoi M, Uchino M, Yamane M, Ozawa N, Fukui M, Tsubota K (2017). In vivo confocal microscopy evaluation of ocular surface in ocular chronic graft-versus-host disease. Sci Rep.

[16] Aketa N, Yamaguchi T, Asato T, Yagi-Yaguchi Y, Suzuki T, Higa K, Kurihara T, Satake Y, Tsubota K, Shimazaki J (2017). Elevated aqueous cytokine levels in eyes with ocular surface diseases. Am J Ophthalmol.

